# Combination of organic acids and low-dose gamma irradiation as antimicrobial treatment to inactivate Shiga toxin-producing *Escherichia coli* inoculated in beef trimmings: Lack of benefits in relation to single treatments

**DOI:** 10.1371/journal.pone.0230812

**Published:** 2020-03-26

**Authors:** Mariana Cap, Celeste Cingolani, Carla Lires, Marina Mozgovoj, Trinidad Soteras, Adriana Sucari, Jimena Gentiluomo, Adriana Descalzo, Gabriela Grigioni, Marcelo Signorini, Celina Horak, Sergio Vaudagna, Gerardo Leotta

**Affiliations:** 1 Instituto Nacional de Tecnología Agropecuaria (INTA), Instituto Tecnología de Alimentos; Buenos Aires, Argentina; 2 Centro Atómico Ezeiza, Comisión Nacional de Energía Atómica (CNEA), Buenos Aires, Argentina; 3 División Higiene y Seguridad Alimentaria y Ambiental, Laboratorio de Alimentos Stamboulian, Buenos Aires, Argentina; 4 Instituto Nacional de Tecnología Agropecuaria (INTA), Estación Experimental Agropecuaria Rafaela, Buenos Aires, Argentina; 5 Facultad de Ciencias Veterinarias UNLP, IGEVET—Instituto de Genética Veterinaria “Ing. Fernando N. Dulout” (UNLP-CONICET LA PLATA), La Plata, Argentina; CINVESTAV-IPN, MEXICO

## Abstract

The aim of this study was to assess the efficacy of lactic acid (LA), caprylic acid (CA), high- (HDI) and low- (LDI) dose gamma irradiation and LDI combined with LA or CA on the inactivation of a pool of Shiga toxin-producing *Escherichia coli* (STEC) strains inoculated on beef trimmings. The three most efficacious treatments were selected to study their effect on meat quality parameters and sensory attributes. The inoculum included five native STEC serogroups (O26, O103, O111, O145 and O157). The treatments applied were 0.5% LA, 0.04% CA, 0.5 kGy LDI, 2 kGy HDI, LDI+LA and LDI+CA. Beef trimmings were divided into two groups; one was inoculated with high (7 log CFU/g) and the other with low (1 log CFU/g) level of inoculum. Efficacy was assessed by estimating log reduction and reduction of *stx-* and *eae-*positive samples after enrichment, respectively. Results showed that treatments with organic acids alone were not effective in reducing STEC populations. For high inoculum samples, the most effective treatment was HDI followed by LDI+LA and LDI alone or combined with CA. For low inoculum samples, the most effective treatment was HDI followed by LDI alone or combined with organic acids. Concerning meat quality parameters and sensory attributes, irradiation treatments (LDI and HDI) caused minimal changes, while LDI+LA modified them significantly compared with the control. Therefore, based on our results, no benefits were observed after combining organic acids with gamma irradiation.

## Introduction

Shiga toxin-producing *Escherichia coli* (STEC) are foodborne pathogens that can cause bloody diarrhea, hemorrhagic colitis and hemolytic uremic syndrome (HUS). In Argentina, STEC serogroups O157, O26, O103, O111, O145 and O121 have been classified as adulterant in ground beef. In the United States (US), serogroups O157, O26, O45, O103, O111, O121 and O145 are recognized as adulterant in ground beef and beef trimmings [1]. In the European Union, detection of the six major STEC serogroups (O157, O26, O103, O111, O145 and O104:H4) in sprouts is mandatory [2].

Beef trimmings are pieces of meat remaining after steaks, roasts and other cuts are removed, and which are often used to make ground beef or hamburgers. A study evaluating the prevalence of non-O157 STEC in beef carcasses, cuts and trimmings from eight Argentinean abattoirs showed that 5.8% pools of carcass and cut samples and 7% pools of beef trimming samples were positive for non-O157 STEC [3]. These findings demonstrated that beef trimmings were an important vehicle of STEC and reflected the importance of applying antimicrobial interventions. In this context, chemical and physical interventions have been evaluated with diverse results [4].

Among chemical interventions, organic acids are by far the most frequently used decontaminants, particularly lactic acid (LA). Studies on the decontamination potential of LA showed that it effectively reduced *E*. *coli* O157:H7 and STEC strains more than 1 log CFU/g [5,6]. Currently, a new decontamination approach based on caprylic acid (CA) is used; this is a natural, 8-carbon, medium-chain fatty acid present in breast milk, bovine milk and coconut oil [7,8]. According to the joint Food and Agriculture Administration (FAO)/World Health Organization (WHO) Expert Committee on Food Additives (JECFA), CA is safe when used as flavor [9]. In the US, CA has been approved for application on ready-to-eat meat and meat products as long as it does not exceed 400 ppm by weight of the finished food product [10]. Studies carried out in beef trimmings inoculated with *E*. *coli* O157:H7 and processed with different organic acids showed that 30 000 ppm CA was highly effective [11].

Among physical interventions, gamma irradiation is a well-known method for controlling microorganisms. Gamma irradiation doses ≤ 2.5 kGy have been reported to cause 5 log CFU/g reductions of non-pathogenic *E*. *coli* O157:H7 (NCTC 12900) inoculated on beef trimmings [12]. The safety and wholesomeness of food irradiation have been officially endorsed by international organizations such as the WHO, FAO and the International Atomic Energy Agency (IAEA).

The combination of chemical and physical treatments has also been explored. For instance, the combined antimicrobial effect of propionic, lactic and acetic acid as pre-sensitization to low-dose gamma irradiation (LDI; 1, 2 and 3 kGy) on *Bacillus cereus* in sheep/goat meat was higher as compared with the individual treatments [13]. Similarly, combined treatments involving lactic, citric and acetic acids and LDI (1, 2 and 3 kGy) were useful to extend the shelf life of pork loins during post-irradiation storage [14]. Even though the objective of antimicrobial treatments is to eliminate pathogens, their effect on food quality should also be considered.

The aims of the present study were to assess the efficacy of organic acids and gamma irradiation, either alone or combined, to inactivate a pool of STEC strains inoculated on beef trimmings, to identify the most effective treatments and evaluate their effect on meat quality parameters and sensory attributes.

## Materials and methods

### Experimental design

A completely randomized design was applied to evaluate the efficacy of individual or combined treatments to inactivate STEC-inoculated beef trimmings. Seven treatments (details are provided in the Chemical Treatments and Irradiation Treatments sections) were evaluated and the design was applied six times with five independent units. Sample size was enough to detect at least 0.8 ± 1.0 log CFU/g differences (95.0% confidence interval [CI]) in STEC counts of the high inoculum experiment, and at least 20% reduction in serogroup prevalence (95.0% CI) of the low inoculum group.

For meat quality evaluation and sensory analysis, beef burgers were prepared with beef trimmings exposed to the three most effective treatments in terms of STEC log reductions. All experiments were carried out three times in duplicate (two burgers). For sensory analysis, pairwise sample presentation was chosen for color assessment. Accordingly, samples were presented frozen in a monadic sequential order and a balanced block design was used to avoid presentation bias. A triangle test was conducted to evaluate overall flavor using a balanced block design with the following parameters: α = 0.05, β = 0.20 and pd = 30% [15,16].

#### Bacterial strains and inoculum preparation

For this study, we used STEC strains O26 (*stx*_1_/*eae*) and O157 (*stx*_2_/*eae*) isolated from beef products, O145 (*stx*_2_/*eae*) isolated from a patient with HUS, and O103 (*stx*_1_/*eae*) and O111 (*stx*_2_/*eae*), both isolated from patients with diarrhea. The strains were kept in frozen culture at -80 ºC. Then, subcultures were prepared by inoculating a test tube containing 10 ml tryptic soy broth (TSB, Biokar, France) with a single colony grown in tryptic soy agar (TSA, Biokar, France). Cultures were individually incubated at 37°C overnight. Cells were harvested by centrifugation at 4000 x g for 10 min. The pellets were washed twice with phosphate-buffered saline (PBS, pH 7.2, Oxoid, UK). The pool of strains was prepared by mixing equal volumes of each strain in PBS.

#### Sample preparation and inoculation procedure

Beef trimmings were obtained from “Frigorífico Gorina” (34°54´29”S 58°02´25”O), a local slaughterhouse. After freezing, they were irradiated at 10 kGy to eliminate the interference of local microbiota. Upon arrival to the laboratory, trimmings were divided into samples of 25 g each and placed into stomacher bags. For the inoculation procedure, 50 μl of a STEC pool were added to the high inoculum samples to obtain a final concentration of 7 log CFU/g, and 50μl of a diluted STEC pool were added to the low inoculum samples to obtain a final concentration of approximately 1 log CFU/g.

#### Chemical treatments

The chemical treatments applied were 0.5% LA (Purac 85%, Netherlands) and 0.04% CA (Sigma Chemical Co, USA), both at 50°C. Antimicrobials (w/w) were added undiluted and mixed by pressing the bag externally. Samples were kept at 4°C until chemical analysis or gamma irradiation.

#### Irradiation treatments

Gamma irradiation was performed with two doses: 0.5 kGy (low-dose irradiation, LDI) and 2 kGy (high-dose irradiation, HDI). Treatments were carried out in a semi-industrial irradiation facility (cobalt-60 source) at the Centro Atómico Ezeiza, Comisión Nacional de Energía Atómica, Argentina (activity, 820 kCi; temperature, 12 ± 0.5°C; average dose rate, 8.7 kGy/h; average dose uniformity, 1.05 kGy). Electron paramagnetic resonance (E-scan Bruker) with BioMax^TM^ alanine dosimeter film (Kodak) was used to measure the absorbed dose. The calibration curve was provided and is traceable to the primary laboratory NIST (National Institute of Standards and Technology, USA).

#### Microbiological analysis of high- inoculum samples

A total of 225 ml of 0.1% peptone water (PW, Biokar, France) was added to the sample into the bag. Immediately afterwards, samples were stomached (easy Mix, AES, France) for 60 s and serial dilutions were prepared. STEC counts were performed in TSA and MacConkey agar (MAC, Biokar, France). A duplicate set of plates was incubated overnight at 37°C.

Log reductions were calculated by subtracting STEC counts in TSA of treated samples from STEC counts in TSA of control samples. Injured cells were calculated as the difference in microbial counts between TSA and MAC.

#### Microbiological analysis of low-inoculum samples

A total of 225 ml of modified TSB (mTSB, Biokar, France) was added to the sample into the bag, which was then incubated at 42°C for 20 h. After the enrichment step, samples were tested for the presence of *stx*_1_, *stx*_2_ and *eae* genes by real-time polymerase chain reaction (RT-PCR, Pall Corporation, USA). Samples positive for *stx* and *ea*e genes were tested for the presence of serogroup molecular markers by RT-PCR (Pall Corporation, USA).

#### Meat quality parameters

As already mentioned, meat quality parameters were evaluated in beef burgers prepared with beef trimmings exposed to the three most effective treatments in terms of STEC log reductions.

*Preparation of beef burgers*. Beef trimmings were obtained from a local slaughterhouse. Upon arrival to the laboratory, samples were divided into four batches. One was used as control and the other three were used for the preparation of irradiated- and/or chemically-treated samples. Beef trimmings were ground twice through a plate with 4-mm hole openings to ensure uniformity. Temperature was monitored preventing it from rising beyond 10°C. Sodium tripolyphosphate (STPP, N 15–16 Chemische Fabrik Budenheim R.A Oetker, Budenheim) was added and manually mixed. Then, salt (Dos Anclas, Argentina) previously dissolved in water at 8°C was added and manually mixed for 5 min. Burgers were formulated with 90% beef trimmings (12% fat), 2% NaCl, 0.25% STPP and 7.75% tap water; they were hand-pressed, placed into a 100-mm mold and frozen at -20°C until further analysis. Good handling practices were followed during burger preparation.

*pH determination*. For pH determination, a 10 g aliquot of raw burger was homogenized with 90 ml of chilled distilled water and measured with a digital pH meter (Thermo Orion model 420, USA) equipped with a combination pH electrode (Thermo Orion Model 8102BN ROSS Electrode, Beverly MA, USA) and an ATC-Probe (Thermo Orion, Beverly MA, USA). The electrode was calibrated immediately before measurement using four buffer solutions at pH 7.0.

*Color measurements*. Color determinations were carried out using a Minolta CR-400 chroma meter (Konica Minolta, Japan) following the recommendations of the American Meat Science Association (AMSA) [17], with D65 illuminant and 2° observer. The CIE L*a*b* system was used to obtain the values of three chromatic parameters: L* (black–white component, lightness), a* (redness/greenness) and b* (yellowness/blueness). Chroma (C*, saturation) and the hue angle (h°, tone) were calculated as follows: C = (a*2 + b*2)^0.5^ and h° = arctg (b*/a*). Hue angle is the change in color from red to yellow, with higher values representing less red product. Raw burgers were allowed to bloom for 45 min prior to the first measurement. Six scans from each sample were averaged for statistical analysis.

*Cooking weight loss (CL)*. Cooking weight loss was determined by measuring the sample weight before and after heat treatment, and reported as the percentage of weight loss with respect to the initial value. Samples were cooked in an electric grill (Spectrum Brands George Foreman, USA) at 155°C. Sample temperature was monitored with a K-thermocouple inserted in the geometric center of the sample until it reached an internal temperature of 74°C. Data were recorded using a digital multimeter (Fluke model Hydra 2620A).

*Kramer shear test*. The instrumental texture of beef burgers was estimated with a 10-blade Kramer-shear cell connected to a texture analyzer (model TA.XT plus, Stable Micro Systems, UK). A load cell of 50 kg with a crosshead speed of 3.3 mms^-1^ was used. From each cooked burger, four parallelepipeds (15 x 60 mm, 10 mm high) were measured perpendicularly to the blades, recording maximum shear force (N) and work of shearing (J).

*Lipid oxidation measurement*. Lipid oxidation was analyzed with the thiobarituric acid reactive substances (TBARS) assay using the technique described by Descalzo et al. [18] with slight modifications. Two grams of each sample were homogenized (Polytron, Kinematica, Switzerland) with 6.25 mL of trichloroacetic acid (2.8%) and 6.25 mL distilled water for 20 s. Slurry was left to filter through a Whatman N°1 filter paper and duplicate samples of filtrate (1 mL) were added to an equal volume of 0.02M TBA. An equal volume of distilled water was added to the third replicate to act as a turbidity blank for each sample. Samples were vortexed for 10 s, incubated in a water bath at 70°C for 1 h until pink color development and allowed to cool for 10 min. Absorbance was read at 532 nm. TBARS were calculated using 1,1,3,3–151 tetramethoxypropan (TEP) as standard. Results were expressed as mg of malondialdehyde (MDA) equivalents/kg of dry matter (DM). TBARS were calculated as follows:

$$TBARS\;number\;\left(mg\;eq\;MDA/kg\;DM\right)=OD_{532}\;x\;1/slope\;\left(mol\right)\;x\;72.06\;g\;MDA\;\left(mol^{‐1}\right)/g\;sample\;x\;85/100\;x\;1.\;10^{6}$$

where OD is the optical density at 532 nm, TEP is used as standard for MDA equivalents 1 mol TEP = 1 mol MDA reacting with TBA; 72.06 g/mol is the molecular weight of MDA, with a recovery of 85% for the system and 1.10^6^ conversion mg/kg.

*Sensory analysis*. Sensory tests were carried out to assess perceptible differences in color and flavor among treated and control (burgers made from untreated beef trimmings) samples.

*Color assessment*. A difference-from-control (DFC) test with a blind control was developed to assess significant differences in raw burger color among treated and control samples. This test is used to estimate the magnitude of difference between a treated sample and a standard control and is useful when there is inherent product variability [19–21]. Sensory color assessment was carried out under standard lightning conditions using a light cabinet (Verivide, CAC 120, UK) and illuminant D65. The panel was formed by 20 untrained assessors recruited from the Food Research Institute. The viewing angle was kept constant (45°) to the light source throughout the study [22]. Assessors were asked to compare the color of each codified sample in relation to a fixed control sample and to record the magnitude of the difference perceived on a ballot paper. A numerical rating scale with verbal anchors was used, where -3 = much weaker than the control sample; -2 = moderately weaker than the control sample; -1 = slightly weaker than the control sample; 0 = no difference in color; 1 = slightly stronger than the control sample; 2 = moderately stronger than the control sample; and 3 = much stronger than the control sample.

*Overall flavor assessment*. The triangle test was used to assess overall flavor differences among treated and control samples [15]. Frozen burgers were cooked for 9 min in a preheated electric grill (200 ± 5°C) (Spectrum Brands George Foreman, USA) to achieve an internal temperature of 71°C [22]. Internal temperature was verified using a probe-type thermocouple connected to a data acquisition system (Hewlett Packard 39470A). The panel was formed by 20 untrained assessors recruited from the Food Research Institute. Hot burger samples (2 x 2 cm) were served in codified disposable thermal containers and presented in a monadic sequential order. Assessors´ remarks on the reasons for their choice were reported in a comments section.

### Statistical analysis

In the high inoculum experiment, TSA and MAC STEC counts in all treatments (control, LA, CA, LDI + LA, LDI + CA and HDI) were analysed using ANOVA and Student-t test. STEC count reduction in all treatments was analysed using ANOVA.

In the low inoculum experiment, comparison of the presence of *stx* and *eae* genes and molecular markers of serogroups O26, O103, O111, O145 and O157 among treatments was performed using the generalized linear model (GLM). Presence or absence of *stx* and *eae* genes and serogroups were the outcome variables in the GLM, using a binary logistic distribution as linked function.

Meat quality parameters were analysed as described for the high inoculum experiment. For the sensory analysis, comparisons between the control and each of the treated samples were performed using two-way ANOVA (with Dunnet´s multiple comparison as post hoc test).

All statistical analyses were performed using InfoStat software (Universidad Nacional de Córdoba, Argentina).

## Results

### High-inoculum samples

Bacterial counts in samples treated with LA and CA and in control samples (either in TSA or MAC) did not differ significantly. Survival (log CFU/g) of STEC in TSA and MAC of samples treated with LDI, LDI + LA, LDI + CA and HDI differed from control samples and among themselves, except for LDI alone and LDI + CA (p < 0.05). The most effective treatment was HDI, resulting in bacterial counts under the detection limit (2 log CFU/g). The second most effective treatment was LDI + LA (log reduction, 1.68 log CFU/g). Finally, the third most effective treatment was LDI alone or combined with CA (average log reduction, 1.38 log CFU/g). None of the treatments, either individual or combined, caused a significant number of injured cells (Table 1).

**Table 1:** Antimicrobial treatment effects on bacterial count reduction (log CFU/g) of beef trimmings inoculated with STEC by plating on triptic soy agar (TSA) and MacConkey agar (MAC).

Antimicrobial treatments	Bacterial counts in TSA (log UFC/g) Mean (SD)	Bacterial count reduction (log CFU/g)	Bacterial counts in MAC (log CFU/g) Mean (SD)	Injured cells (log CFU/g)
**Control**	7.14 (0.11) a	-	6.56 (0.27) a	-
**LA**	7.01 (0.10) a	0.13	6.57 (0.31) a	0.44
**CA**	7.13 (0.26) a	0.01	6.75 (0.27) a	0.38
**LDI**	5.75 (0.34) b	1.39	5.40 (0.26) b	0.35
**LDI + LA**	5.46 (0.50) c	1.68	5.01 (0.58) c	0.45
**LDI + CA**	5.77 (0.29) b	1.37	5.43 (0.34) b	0.34
**HDI**	ND	> 5	ND	-

Results are expressed as mean (SD); n = 30 per treatment.

a, b, c Interventions with no common letter differed significantly (*P* < 0.05; one-way ANOVA).

LA, lactic acid (0.5%); CA, caprylic acid (0.04%); LDI, low-dose irradiation (0.5 kGy); HDI, high-dose irradiation (2 kGy).

ND: not detected, counts were below the limit of detection (2 log CFU/g).

### Low-inoculum samples

Results of samples inoculated with a low bacterial concentration are shown in Table 2. All samples (100%) treated with LA and CA were positive for *stx* and *eae* genes by RT-PCR after enrichment. Irradiation, either alone or combined with organic acids, was effective in reducing the percentage of samples positive for *stx* and *eae* genes (70% in average). The most effective treatment was HDI, as none of the treated samples was positive for *stx* or *eae* genes. Regarding the percentage of samples positive for STEC serogroups, no statistical differences were found among control samples and samples treated with LA and CA. In samples treated with LDI alone or combined with LA or CA, the percentage of positive samples for STEC serogroups differed from the control samples, but not among themselves (p < 0.05).

**Table 2:** Effect of intervention on the prevalence (%) of *stx* and *eae* genes and molecular markers of serogroups O26, O103, O111, O145 and O157.

Antimicrobial treatments	*stx* and *eae(%*	O26	O103	O111	O145	O157
**Control**	100 ^a^	100 ^a^	100 ^a^	100 ^a^	97 ^a^	97 ^a^
**LA**	100 ^a^	97 ^a^	100 ^a^	93 ^a^	89 ^a^	100 ^a^
**CA**	100 a	100 ^a^	97 ^a^	97 ^a^	100 ^a^	100 ^a^
**LDI**	70 ^b^	23 ^b^	47 ^b^	23 ^b^	30 ^b^	23 ^b^
**LDI + LA**	63 ^b^	27 ^b^	40 ^b^	37 ^b^	30 ^b^	33 ^b^
**LDI + CA**	77 ^b^	27 ^b^	43 ^b^	23 ^b^	27 ^b^	27 ^b^
**HDI**	ND	ND	ND	ND	ND	ND

^a, b^ Interventions with no common letter are significantly different (*P* < 0.05) using Generalized Lineal Model.

LA, lactic acid (0.5%); CA, caprylic acid (0.04%); LDI, low-dose irradiation (0.5 kGy); HDI, high-dose irradiation (2 kGy).

ND: not detected.

### Meat quality parameters

In terms of STEC inactivation, the best three treatments were HDI, LDI + LA and LDI alone. Results of meat quality parameters after these selected treatments are shown in Table 3.

**Table 3:** Effect of interventions (LDI, LDI combined with LA and HDI) on the meat quality parameters of beef burgers.

Meat quality parameters		Antimicrobial treatments Mean (S.D.)			
		Control	LDI	LDI + LA	HDI
pH of raw beef burgers		6.00 (0.03) ^a^	5.91 (0.03) ^b^	5.02 (0.02) ^c^	5.91 (0.02) ^b^
**Chromatic parameters of raw beef burgers**	L*	41.92 (0.89) ^a^	43.11 (1.85) ^a^	41.47 (1.67) ^a^	42.13 (1.73) ^a^
	a*	14.49 (1.13) ^a^	12.51 (0.95) ^b^	9.50 (0.69) ^c^	13.58 (1.35) ^a^^b^
	b*	13.00 (0.56) ^a^	12.62 (0.65) ^a^^b^	12.06 (0.50) ^b^	12.45 (0.43) ^a^^b^
	C*	19.49 (1.12) ^a^	17.80 (1.10) ^b^	15.37 (0.79) ^c^	18.46 (1.22) ^a^^b^
	h°	42.02 (1.65) ^c^	45.42 (1.08) ^b^	51.83 (1.26) ^a^	42.73 (2.29) ^c^
**Chromatic parameters of cooked beef burgers**	L*	41.79 (2.49) ^b^	40.68 (3.27) ^b^	46.80 (2.49) ^a^	41.55 (3.81) ^b^
	a*	7.66 (0.61) ^a^^b^	8.17 (0.54) ^a^	6.87 (0.81) ^b^	7.93 (0.61) ^a^
	b*	11.10 (0.34) ^a^	10.72 (0.33) ^a^	9.27 (1.19) ^b^	10.67 (0.40) ^a^
	C*	13.51 (0.51) ^a^	13.49 (0.48) ^a^	11.55 (1.39) ^b^	13.31 (0.61) ^a^
	h°	55.48 (2.04) ^a^	52.79 (1.70) ^b^	53.34 (1.81) ^a^^b^	53.50 (1.93) ^a^^b^
**Cooking weight loss (%)**		16.30 (1.14) ^b^	18.58 (2.62) ^b^	31.23 (2.79) ^a^	18.82 (2.67) ^b^
**Kramer shear test**	SF (N)	183.55 (12.31) ^a^^b^	170.42 (18.88) ^b^	202.18 (18.89) ^a^	189.54 (21.36) ^a^^b^
	WS (J)	1.31 (0.09) ^a^	1.23 (0.14) ^a^	1.37 (0.20) ^a^	1.37 (0.22) ^a^
**Lipid oxidation**	TBARS (mg eq MDA /kg DM)	0.53 (0.04) ^d^	0.87 (0.07) ^b^	1.23 (0.09) ^a^	0.74 (0.03) ^c^

L*: lightness; a*: redness; b*: yellowness; C*: chromaticity; h°: hue angle.

^a, b, c, d^ Interventions with no common letter differed significantly (*P* < 0.05) using one-way ANOVA.

LA, lactic acid (0.5%); LDI, low-dose irradiation (0.5 kGy); HDI, high-dose irradiation (2 kGy).

SF, maximum shear force (N); WS, work of shearing (J); TBARS, thiobarbituric acid reactive substances.

### pH

In treated samples, pH values differed from those measured in control samples. In the latter, pH was 6, similar to that found in samples treated with LDI or HDI alone (5.91). In samples treated with LDI + LA, pH was 5.02, statistically different from that measured in control, LDI and HDI-treated samples (Table 3).

### Color measurements

Raw samples treated with LDI alone exhibited lower redness and intense color (< a* and C*), higher hue-angle (> h°) but the same lightness (= L*) as raw control samples (p > 0.05). Raw samples treated with LDI + LA exhibited the lowest redness and intensity (<< a*, and C*), the highest hue-angle (>>h) and the same lightness (= L*) as control, LDI- and HDI-treated samples; yellowness was also lower than in control samples (< b*), but not significantly different from that observed in samples treated with LDI and HDI alone. Overall, raw samples treated with HDI alone did not differ from control samples in any of the parameters analyzed (p < 0.05). Samples of cooked burgers treated with LDI or HDI alone exhibited similar values of chromatic parameters (same redness, yellowness, color intensity, hue-angle and lightness) as control samples (= a*, b*, C*, h° and L*). Cooked samples treated with LDI + LA exhibited higher lightness (> L*) but lower yellowness and intensity (< b* and C*) than control, LDI- and HDI-treated samples (Table 3).

### Cooking weight loss

Samples treated with LDI + LA showed significantly increased CL compared with control, LDI- and HDI-treated samples. On the other hand, CL was not significantly different in samples irradiated with LDI or HDI alone as compared with control samples (p < 0.05) (Table 3).

### Kramer shear test

Instrumental texture results of samples treated with LDI and HDI did not differ from those obtained in control samples. Shear force results were higher in LDI + LA-irradiated samples than in samples irradiated with LDI alone (p < 0.05). No differences were observed in work of shearing among treatments (p > 0.05) (Table 3).

### Lipid oxidation

Irradiation and the acid environment increased the effect of lipid oxidation. In irradiated samples, lipid oxidation was different from that of control samples and among treatments (p < 0.05). TBARS values of samples irradiated with LDI + LA was 1.23 mg eq MDA/kg DM, followed by LDI (0.87 mg eq MDA/kg DM) and HDI (0.74 mg eq MDA/kg DM). As expected, control samples presented the lowest lipid oxidation (0.53 mg eq MDA/kg DM).

### Sensory analysis

#### Color assessment

The resulting DFC scores were compared with DFC data from the blind control, calculating the difference by subtracting the DFC scores of the blind control sample from those of the test sample. A significant difference was found between the reference and the treated samples (p < 0.0001). The Dunnett’s test showed that DFC scores of the three treated samples were significantly different from the blind control (*P* < 0.05). Color intensity was slightly to moderately stronger in LDI- and HDI-treated samples than in the control, and moderately to much stronger in LDI + LA-treated samples compared with the control (Table 4).

**Table 4:** Effect of interventions (LDI, LDI combined with LA and HDI) on mean panel data and *P*-value of the difference from control (DFC) test of beef burgers.

Antimicrobial treatment	Mean DFC	Mean Test DFC Minus Mean Blind Control DFC	*P-*Value
Control	0.28		
LID	1.50	1.23	<0.0001
LID + LA	2.83	2.55	<0.0001
HID	1.73	1.45	<0.0001

LA, lactic acid (0.5%); LDI, low-dose irradiation (0.5 kGy); HDI, high-dose irradiation (2 kGy).

#### Overall flavor

Results of the triangle test were as follows: out of the 40 samples treated with LDI alone, 20 were identified as different from the reference; in samples treated with LDI+LA, 34 were identified as different from the reference, and among samples treated with HDI, 24 were identified as different from the reference (*P* < 0.001 in all cases). The main attributes responsible for such differences were juiciness, saltiness and texture. In relation to the latter, samples treated with LDI + LA presented a visible lack of cohesiveness. No off-flavors were detected in irradiated samples.

## Discussion

Here we report the effects of gamma irradiation and organic acids, either applied individually or combined, on STEC inactivation and meat quality parameters. While our results showed that organic acids alone were not effective to inactivate a pool of STEC strains inoculated on beef trimmings, other authors have reported the efficacy of CA to reduce pathogenic microbial counts in meat products. For example, a 2-log CFU/g reduction of *Listeria monocytogenes* after treating minced meat with 0.5% CA has been reported [23]. However, the concentration used by the cited authors was more than 10 times higher than the one used in the present study (0.04%) and it exceeded the concentration recognized as safe by USDA for use in meat products [10]. Mohan & Pohlman [11] reported a 1.1 log CFU/g reduction of *E*. *coli* (ATCC 25922) after treating beef trimmings with 0.04% CA and subsequently mixing with 10% (w/v) trisodium phosphate (TSP). The difference between these results and ours could be due to the combined effect of CA and TSP as well as to a different strain resistance. Regarding LA treatment, our results are in agreement with those reported by Harris, Brashears, Garmyn, Brooks, & Miller [24], who informed that 2 and 5% LA showed no measurable reduction of *E*. *coli* inoculated on the surface of beef trimmings. Conversely, Ransom et al. [5] reported that 2% LA reduced 1.1 log CFU/g of *E*. *coli* O157:H7 inoculated on beef trimmings. Differences among assays may be due to differences in strain resistance, acid concentration and/or experimental conditions.

The most effective treatment was HDI; total STEC inactivation was achieved in samples with high as well as low inoculum. These results are in agreement with already published data [12,25]. In high inoculum samples, the second most effective treatment was LDI with 0.5% LA, as described by other authors. For instance, Bhide et al. [13] reported that presensitization with 2% LA followed by 1, 2 and 3 kGy in sheep/goat meat reduced the total viable counts and *Bacillus cereus* population more than irradiation alone. Kim, Jang, Lee, Min & Lee [14] evaluated the combined effects of 2% LA and electron-beam irradiation (1, 2 and 3 kGy) on total aerobic bacterial counts and coliform counts of naturally contaminated pork loins, showing that the combined treatment was more effective than either treatment alone. In this work, the pH value may have influenced the increase in bactericidal effect (5.02 in samples treated with LDI + LA, and 5.91 in samples treated with LDI alone). The same phenomenon was reported by Bhide et al. [13] and Surve, Sherikar, Bhilegaonkar, & Karkare [26]. In low inoculum samples, the higher efficacy of LDI with 0.5% LA treatment was not observed. Differences between the results obtained with high and low inoculum samples may be due to the fact that the damage caused by LA was not enough to cause complete cell inactivation and cells were therefore able to survive and recover during the enrichment procedure. The effect of population size on *E*. *coli* O157:H7 log reductions after treatments was also described by Koseki, Yoshida, Kamitani, & Itoh [27], who demonstrated the importance of assessing treatment efficacy with more than one level of inoculum. While the high inoculum level is used to estimate log reductions and to compare efficacy among treatments, the low inoculum level is used to mimic natural bacterial contamination. In the latter case, the treatment is effective when it is capable of inactivating all bacteria present in the sample.

The third most effective treatment in samples with high inoculum was LDI either alone or combined with 0.04% CA, the log reduction achieved in the present study was of 1,3 log CFU/g. This result is in agreement with Sommers et al. [25], who informed that the radiation dose needed to obtain 1 log reduction (90%) of STEC inoculated on ground meat ranged from 0.16 to 0.48 kGy. However, the reduction was lower than that reported by Xavier et al. [12] (2.6 log CFU/g reduction in non-pathogenic *E*. *coli* O157:H7 inoculated on beef trimmings after treatment with 0.5 kGy). The difference between our results and those previously cited may be attributed to the nature of the strain used in each assay, since the mentioned authors worked with only one non-pathogenic *E*. *coli* strain [12] while we used a pool of five STEC strains, all positive for *eae* genes. Previous studies have reported a higher resistance of positive-*eae E*. *coli* strains to gamma irradiation as compared with negative ones [25]. Besides, differences could also be due to the composition of the food matrices as well as to the gamma irradiation equipment used in each case. In low inoculum samples treated with LDI either alone or combined with 0.04% CA, the percentage of *stx-* and *eae-*positive samples was 70%. The percentage of positive samples for serogroup molecular marker after treatment and enrichment was in average 25%, except for O103 (47%). The lower percentage of positive signal for serogroup molecular markers compared with the positive signal for *stx* and *eae* genes was expected, as the number of initial bacteria for each serogroup was much lower than the number of initially *stx-* and *eae-*positive bacteria. In the case of O103, the difference with the other serogroups was not expected and might be due to a higher resistance of the strain. Although 0.5 kGy LDI did not manage to achieve total STEC inactivation, this was not the intended goal of the treatment, which was aimed at determining whether there was synergist effect between gamma irradiation and organic acids.

As to meat quality parameters, minimal changes were observed between irradiated (LDI-HDI) and control samples and between LDI- and HDI-treated samples. Raw irradiated samples presented lower redness and intensity but higher hue-angle than control samples. Similar findings were reported by Xavier et al. and Nanke et al. who treated beef with 2 and 1.5 kGy, respectively [12,28]. These changes were also observed in the sensory analysis, where color intensity was described as slightly to moderately stronger than in the control. Changes may be due to the normal process by which myoglobin undergoes oxidation, which is accelerated by the rapid generation of large amounts of metmyoglobin when irradiation is conducted in oxygen-containing environments [29]. Regarding lipid oxidation, irradiated samples registered higher TBARS values than control samples, which was expected because irradiation favors the propagation of fatty acid free radicals and the formation of oxygen free radicals. Surprisingly, HDI showed lower TBARS value than LDI. This was unexpected, considering that in many reports the oxidative status of irradiated meat increased proportionally to the irradiation dose. This could be explained by the fact that MDA and other short chain carbon products of lipid oxidation are not stable for a long period of time. This is because oxidation of these products yields organic alcohols and acids, which are not determined by the TBA test [30]. However, it is still the most used, especially for meats, because the compounds with the greatest interference can be aldehyde of the carbohydrates and the meat has very little carbohydrates. Regarding, cooking weight loss and texture profile they remained the same as in control samples. Finally, flavor assessment showed no off-flavors, even though treated samples were different from control samples.

Regarding meat quality of LDI + LA-treated samples, changes were significant as compared to control samples. Raw samples treated with LDI+LA showed the lowest redness, yellowness and intensity, the highest hue-angle and the same lightness than control samples. As these differences were not observed in samples treated with LDI alone, we assumed that LA treatment was responsible for them. These results are in agreement with those reported by other authors [31,32]. Such differences were also perceived in the sensory analysis by assessors, who described color intensity as moderately to much stronger than in the control sample. Regarding cooking weight loss, values were higher than those registered in control samples, as also shown by Friedrich et al. [34]. This phenomenon may also be due to the decrease in pH as well as the protein denaturation induced by LA, which resulted in a decreased water holding capacity of proteins [33]. Concerning lipid oxidation, TBARS values of samples treated with LDI+LA and LDI alone were higher than in control samples. Higher lipid oxidation could be attributed either to the cumulative effect of irradiation and organic acid or to the lower pH values [14,34]. Regarding flavor, even though assessors described treated samples as different from control samples, no off-flavors were detected. The same observation applied to samples treated with irradiation alone.

## Conclusions

Organic acid treatments alone were not effective as far as the inactivation of STEC was concerned. The most effective treatment was high dose gamma irradiation (HDI; 2 kGy) since it achieved total STEC inactivation in high and low inoculum samples and caused minimal changes in meat quality parameters and sensory attributes. After treatment with low dose gamma irradiation (LDI; 0.5 kGy) alone or combined with 0.04% caprylic acid, STEC lethality was 1.38 log CFU/g in high inoculum samples and caused 30% reduction in the number of samples positive for *stx-* and *eae-*genes in low inoculum samples. After treatment with LDI combined with 0.5% lactic acid, STEC lethality was higher than with LDI alone (1.68 log CFU/g) in samples with high inoculum, but it remained the same in samples with low inoculum (30%). Concerning meat quality parameters and sensory attributes, treatment with LDI and 0.5% LA was less effective than LDI alone in preserving these parameters. Therefore, based on our results, no benefits were observed after combining organic acids with gamma irradiation.

## Supporting information

S1 Table STEC counts on triptic soy and MacConkey agar. (XLSX)

## References

[1] FSIS. Shiga toxin-producing *Escherichia coli* in certain raw beef products. 2012. Available: https://www.federalregister.gov/documents/2011/11/23/2011-30271/shiga-toxin-producing-escherichia-coli-in-certain-raw-beef-products

[2] EFSA. Scientific Opinion on VTEC-seropathotype and scientific criteria regarding pathogenicity assessment. EFSA J. 2013;11: 3138 10.2903/j.efsa.2013.3138

[3] Brusa V, Restovich V, Galli L, Teitelbaum D, Signorini M, Brasesco H, et al Isolation and characterization of non-O157 Shiga toxin-producing *Escherichia coli* from beef carcasses, cuts and trimmings of abattoirs in Argentina. Chang Y-F, editor. PLoS One. 2017;12: e0183248 10.1371/journal.pone.0183248 28829794 PMC5568767

[4] Sohaib M, Anjum FM, Arshad MS, Rahman UU. Postharvest intervention technologies for safety enhancement of meat and meat based products; a critical review. J Food Sci Technol. 2016;53: 19–30. 10.1007/s13197-015-1985-y 26787929 PMC4711421

[5] Ransom JR, Belk KE, Sofos JN, Stopforth JD, Scanga JA, Smith GC. Comparison of Intervention Technologies for Reducing *Escherichia coli* O157: H7 on Beef Cuts. Food Prot Trends. 2003;23: 24–34.

[6] Kalchayanand N, Arthur TM, Bosilevac JM, Schmidt JW, Wang R, Shackelford SD, et al Evaluation of Commonly Used Antimicrobial Interventions for Fresh Beef Inoculated with Shiga Toxin–Producing *Escherichia coli* Serotypes O26, O45, O103, O111, O121, O145, and O157:H7. J Food Prot. 2012;75: 1207–1212. 10.4315/0362-028X.JFP-11-531 22980002

[7] Jensen RG. The Composition of Bovine Milk Lipids: January 1995 to December 2000. J Dairy Sci. 2002 10.3168/jds.S0022-0302(02)74079-4 11913692

[8] Sprong RC, Hulstein MFE, Van der Meer R. Bactericidal Activities of Milk Lipids Bactericidal Activities of Milk Lipids. Antimicrob Agents Chemother. 2001;45: 1298–1301. 10.1128/AAC.45.4.1298-1301.2001 11257052 PMC90461

[9] JECFA. Evaluation of certain food additive and contaminants. Sixty-third Rep Jt FAO/WHO Expert Comm Food Addit. 2005; 1–226, back cover. Available: http://whqlibdoc.who.int/trs/WHO_TRS_928.pdf

[10] USDA. Safe and Suitable Ingredients used in the Production of Meat, Poultry, and Egg Products. 2015; 1–75. Available: http://www.fsis.usda.gov/wps/wcm/connect/bab10e09-aefa-483b-8be8-809a1f051d4c/7120.1.pdf

[11] Mohan A, Pohlman FW. Role of organic acids and peroxyacetic acid as antimicrobial intervention for controlling *Escherichia coli* O157: H7 on beef trimmings. LWT—Food Sci Technol. 2016;65: 868–873. 10.1016/j.lwt.2015.08.077

[12] Xavier M de la P, Dauber C, Mussio P, Delgado E, Maquieira A, Soria A, et al Use of mild irradiation doses to control pathogenic bacteria on meat trimmings for production of patties aiming at provoking minimal changes in quality attributes. Meat Sci. 2014;98: 383–391. 10.1016/j.meatsci.2014.06.037 25042241

[13] Bhide M., Paturkar A., Sherikar A., Waskar V. Presensitization of microorganisms by acid treatments to low dose gamma irradiation with special reference to Bacillus cereus. Meat Sci. 2001;58: 253–258. 10.1016/S0309-1740(00)00162-5 22062253

[14] Kim BH, Jang A, Lee SO, Min JS, Lee M. Combined effect of electron-beam (beta) irradiation and organic acids on shelf life of pork loins during cold storage. J Food Prot. 2004;67: 168–171. 10.4315/0362-028x-67.1.168 14717368

[15] ISO 4120:2004 IO for S. Sensory analysis—Methodology—Triangle test.

[16] Stone H, Sidel JL. Sensory Evaluation Practices. Sensory Evaluation Practices. 2004 10.1016/B978-012672690-9/50013-5

[17] AMSA AMSA. Meat Color Measurement Guidelines. 2012 10.1007/s11786-018-0341-9

[18] Descalzo AM, Insani EM, Biolatto A, Sancho AM, García PT, Pensel NA, et al Influence of pasture or grain-based diets supplemented with vitamin E on antioxidant/oxidative balance of Argentine beef. Meat Sci. 2005;70: 35–44. 10.1016/j.meatsci.2004.11.018 22063278

[19] Muñoz AM, Civille G V., Carr BT. Sensory Evaluation in Quality Control. New York: Springer Science + Business Media LLC.; 1992.

[20] Kilcast D. Sensory analysis for food and beverage quality control. Sensory Analysis for Food and Beverage Quality Control. 2010 10.1533/9781845699512

[21] Rogers L. Sensory Panel Management: A Practical Handbook for Recruitment, Training and Performance. Sensory Panel Management: A Practical Handbook for Recruitment, Training and Performance. 2017.

[22] AMSA. Research Guidelines for Cookery, Sensory Evaluation, and Instrumental Tenderness Measurements of Meat. American Meat Science Association Educational Foundation 2015 Available: http://www.meatscience.org

[23] Hulankova R, Borilova G, Steinhauserova I. Combined antimicrobial effect of oregano essential oil and caprylic acid in minced beef. Meat Sci. 2013;95: 190–194. 10.1016/j.meatsci.2013.05.003 23743028

[24] Harris D, Brashears MM, Garmyn AJ, Brooks JC, Miller MF. Microbiological and organoleptic characteristics of beef trim and ground beef treated with acetic acid, lactic acid, acidified sodium chlorite, or sterile water in a simulated commercial processing environment to reduce *Escherichia coli* O157:H7 and Salmon. Meat Sci. 2012;90: 783–788. 10.1016/j.meatsci.2011.11.014 22122990

[25] Sommers C, Rajkowski KT, Scullen OJ, Cassidy J, Fratamico P, Sheen S. Inactivation of shiga toxin-producing *Escherichia coli* in lean ground beef by gamma irradiation. Food Microbiol. 2015;49: 231–234. 10.1016/j.fm.2015.02.013 25846936

[26] Surve AN, Sherikar AT, Bhilegaonkar KN, Karkare UD. Preservative effect of combinations of acetic acid with lactic or propionic acid on buffalo meat stored at refrigeration temperature. Meat Sci. 1991;29: 309–322. 10.1016/0309-1740(91)90010-N 22061435

[27] Koseki S, Yoshida K, Kamitani Y, Itoh K. Influence of Inoculation Method, Spot Inoculation Site, and Inoculation Size on the Efficacy of Acidic Electrolyzed Water against Pathogens on Lettuce. J Food Prot. 2003;66: 2010–2016. 10.4315/0362-028x-66.11.2010 14627276

[28] Nanke KE, Sebranek JG, Olson DG. Color characteristics of irradiated aerobically packaged pork, beef, and turkey. J Food Sci. 1999;64: 272–278. 10.1111/j.1365-2621.1998.tb15842.x

[29] Brewer S. Irradiation effects on meat color–a review. Meat Sci. 2004;68: 1–17. 10.1016/j.meatsci.2004.02.007 22062002

[30] Fernández J, Pérez-Álvarez JA, Fernández-López JA. Thiobarbituric acid test for monitoring lipid oxidation in meat. Food Chem. 1997;59: 345–353. 10.1016/S0308-8146(96)00114-8

[31] Friedrich L, Siró I, Dalmadi I, Horváth K, Ágoston R, Balla C. Influence of various preservatives on the quality of minced beef under modified atmosphere at chilled storage. Meat Sci. 2008;79: 332–343. 10.1016/j.meatsci.2007.10.012 22062762

[32] Rodríguez-Melcón C, Alonso-Calleja C, Capita R. Lactic acid concentrations that reduce microbial load yet minimally impact colour and sensory characteristics of beef. Meat Sci. 2017;129: 169–175. 10.1016/j.meatsci.2017.01.007 28324868

[33] Aaslyng MD, Bejerholm C, Ertbjerg P, Bertram HC, Andersen HJ. Cooking loss and juiciness of pork in relation to raw meat quality and cooking procedure. Food Qual Prefer. 2003;14: 277–288. 10.1016/S0950-3293(02)00086-1

[34] Kwoh TL. Catalysts of lipid peroxidation in meats. J Am Oil Chem Soc. 1971;48: 550–555. 10.1007/BF02544560

